# The Complex Interplay between Imbalanced Mitochondrial Dynamics and Metabolic Disorders in Type 2 Diabetes

**DOI:** 10.3390/cells12091223

**Published:** 2023-04-23

**Authors:** Tin Van Huynh, Lekha Rethi, Lekshmi Rethi, Chih-Hwa Chen, Yi-Jen Chen, Yu-Hsun Kao

**Affiliations:** 1 International Ph.D. Program in Medicine, College of Medicine, Taipei Medical University, Taipei 11031, Taiwan; 2 Department of Interventional Cardiology, Thong Nhat Hospital, Ho Chi Minh City 700000, Vietnam; 3 School of Biomedical Engineering, College of Biomedical Engineering, Taipei Medical University, Taipei 11031, Taiwan; 4 International Ph.D. Program for Biomedical Engineering, Taipei Medical University, Taipei 11031, Taiwan; 5 Department of Orthopedics, Taipei Medical University-Shuang Ho Hospital, New Taipei City 23561, Taiwan; 6 School of Medicine, College of Medicine, Taipei Medical University, Taipei 11031, Taiwan; 7 Graduate Institute of Clinical Medicine, College of Medicine, Taipei Medical University, Taipei 11031, Taiwan; 8 Division of Cardiovascular Medicine, Department of Internal Medicine, Wan Fang Hospital, Taipei Medical University, Taipei 11031, Taiwan; 9 Department of Medical Education and Research, Wan Fang Hospital, Taipei Medical University, Taipei 11031, Taiwan

**Keywords:** fission, fusion, metabolic disorders, mitochondrial biogenesis, mitochondrial dynamics, type 2 diabetes mellitus

## Abstract

Type 2 diabetes mellitus (T2DM) is a global burden, with an increasing number of people affected and increasing treatment costs. The advances in research and guidelines improve the management of blood glucose and related diseases, but T2DM and its complications are still a big challenge in clinical practice. T2DM is a metabolic disorder in which insulin signaling is impaired from reaching its effectors. Mitochondria are the “powerhouses” that not only generate the energy as adenosine triphosphate (ATP) using pyruvate supplied from glucose, free fatty acid (FFA), and amino acids (AA) but also regulate multiple cellular processes such as calcium homeostasis, redox balance, and apoptosis. Mitochondrial dysfunction leads to various diseases, including cardiovascular diseases, metabolic disorders, and cancer. The mitochondria are highly dynamic in adjusting their functions according to cellular conditions. The shape, morphology, distribution, and number of mitochondria reflect their function through various processes, collectively known as mitochondrial dynamics, including mitochondrial fusion, fission, biogenesis, transport, and mitophagy. These processes determine the overall mitochondrial health and vitality. More evidence supports the idea that dysregulated mitochondrial dynamics play essential roles in the pathophysiology of insulin resistance, obesity, and T2DM, as well as imbalanced mitochondrial dynamics found in T2DM. This review updates and discusses mitochondrial dynamics and the complex interactions between it and metabolic disorders.

## 1. Introduction

Diabetes mellitus, known as “diabetes”, is a critical challenge for public health and a therapeutic conundrum. Diabetes comprises a collection of metabolic conditions characterized and identified by hyperglycemia due to the deficiency of insulin secretion, impaired insulin action, or both [1]. There are two primary forms of diabetes: type 1 diabetes (T1DM), defined by insulin insufficiency, and immune-mediated pancreatic β-cell destruction. It manifests during the onset of childhood and in the formative phase of adulthood. The most prevalent form of diabetes is type 2 diabetes (T2DM) [2,3]. It is associated with insulin resistance in the early stage, followed by various degrees of β-cell dysfunction, and frequently co-occurs with other metabolic diseases, such as obesity. Diabetes and obesity are two complex multi-factorial, progressive disorders, with obesity the leading independent risk factor for developing T2DM, accounting for more than 90% of diagnoses [2,3]. Before T2DM was clinically diagnosed, there was a significant pancreatic β-cells loss in response to the loss of insulin sensitivity in peripheral tissue, primarily skeletal muscle, adipose tissue, and liver. The remaining β-cells cannot sustain secreting insulin to compensate for insulin resistance at a certain point. The overt T2DM manifests with hyperglycemia and the deterioration of the β-cell mass.

The “powerhouse of the cell,” mitochondria are cellular organelles. The word “mitochondria” comes from two Greek words that denote thread “mitos” and granule “chondros”. Most eukaryotic cells contain highly active mitochondria. It is hypothesized that mitochondria are the descendants of a prokaryote formed in a prehistoric era that underwent an endosymbiotic relationship with early eukaryotes [4]. Translocase of the outer mitochondrial membrane complex, sorting and assembly machinery complex, and porins are three essential protein families in the outer membrane. The oxidative phosphorylation enzyme complexes are located in cristae, formed by folding the inner membrane. The tricarboxylic acid (TCA) cycle is a process that happens in the mitochondrial matrix. The 16.5 kb double-stranded closed circular DNA mitochondrial genome is also found in the matrix. The mitochondrial genome comprises two ribosomal RNAs (12S and 16S rRNA), 13 OXPHOS proteins, 22 transfer RNAs, and 37 genes. Approximately 1500 mitochondrial proteins are encoded by nuclear genes and transported into the mitochondria from the cytoplasm [5].

The dominant role of mitochondria is energy conversion. To sustain bioenergetics and cell energy metabolism, adenosine triphosphate (ATP), which is synthesized by aerobic respiration in the mitochondrion, is required. TCA cycle and oxidative phosphorylation (OXPHOS) are the main series of events involved in the generation of ATP. These molecular mechanisms regulate biological system functions that necessitate the consumption and production of energy, such as integrating multiple metabolic pathways, regulating cellular apoptosis, and perpetuating the calcium homeostasis mechanism [6]. Similarly, mitochondria execute a multitude of roles that contribute to cellular metabolism. Metabolic precursors for macromolecules such as lipids, proteins, DNA, and RNA are produced by mitochondria. Reactive oxygen species (ROS) and ammonia are metabolic byproducts of mitochondria. They also can eliminate or utilize these biological byproducts. The modulation of innate immunity, the regulation of stem cells, the administration of programmed cell death, and aging are all processes in which mitochondria play a significant role. Hence, any potential mitochondrial disruption affects energy homeostasis and regulates cellular metabolism.

The primary dynamic activities include biogenesis (generating two identical healthy mitochondria from a pre-existing one after full growth and completed mitochondrial DNA replication), transportation (directed movement inside a cell), fission (segregation of a single organelle into two heterogenous mitochondria with mtDNA replication), fusion (the combining of two organelles into one), and mitophagy (targeted elimination through the autophagic pathway) [7,8,9,10,11]. Each dynamic process is essential for preserving a robust mitochondrial population, demonstrated to be crucial in both healthy physiology and disease states in organisms ranging from single-celled eukaryotes such as yeast to mammalians [12,13]. Hence, mitochondrial dysfunction can induce the pathogenesis of a wide range of seemingly unrelated disorders [14,15].

“Mitochondrial dysfunction” is caused by a metabolic imbalance of nutrient signal intake, energy production, and/or oxidative respiration [16], which in turn causes mitochondria-associated metabolic disorders. The traditional definition of mitochondrial malfunction is their inability to produce and maintain adequate quantities of ATP [17]. The term is also used to describe the unfavorable physiological reactions of mitochondria to metabolic disturbances, including irregularities in substrate catabolism, calcium buffering, iron transport, mutations in mitochondrial DNA or nuclear mitochondrial genes, changes in mitochondrial dynamics, changes in size and morphology, ROS production, and apoptosis [16,18]. However, the metabolic implications depend heavily on mitochondrial fission and fusion kinetics. Therefore, this review concentrates on the complex interplays between imbalanced mitochondrial dynamics and metabolism in T2DM. 

## 2. Mitochondrial Dynamics: Biogenesis, Transport, Fusion, Fission, and Mitophagy

The ultrastructure of the mitochondrion reflects its biological, cellular, and molecular functions. Mitochondrial dynamics, which regulate the functional equilibrium of the mitochondria, is essential for mitochondrial functioning, energetics, mobility, and its host cell’s fate. Mitochondrial dynamics vary across cell types and tissues, but the critical protein machinery that drives the process has been remarkably preserved through evolution [19]. Reduction and escalation of any of these dynamic processes due to pathological or physiological stressors results in an imbalance affecting mitochondrial function, ultimately leading to multiple disorders in cardiovascular, neurodegenerative, metabolic diseases, and cancer [20,21]. 

### 2.1. Mitochondrial Biogenesis

**Figure 1:**
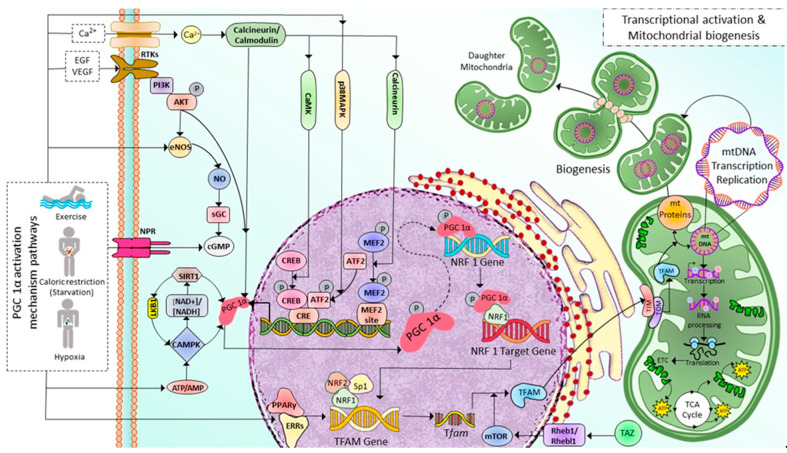
Illustration depicting implicated PGC 1α activation mechanism pathways and associated transcriptional modifications in regulating mitochondrial biogenesis in skeletal muscle cells. Exercise and caloric restriction promote Ca^2+^ entry, tyrosine kinase receptor activation, eNOS production, NPR activation, and decreased ATP/AMP ratio. All these effects, in turn, activate PGC-1α in several mechanisms either by post-translational modifications (deacetylation, phosphorylation) or by regulating PGC-1α transcription. First, PGC-1α increases NRF1 expression, then PGC-1α and NRF1 bind to the target gene and initiate the transcription of the TFAM gene. Exercise also increases the PPARγ-ERRs complexes, which enhance TFAM gene transcription. *Tfam* mRNA is then translated into TFAM protein; this process is facilitated by mTOR, a downstream target of TAZ-Rheb1/Rhebl1 signaling. Finally, TFAM is transported into mitochondria through TOM/TIM complexes, where it induces mitochondrial DNA replication, transcription, and translation leading to mitochondrial biogenesis. Ca^2+^: calcium ion; EGF: Epidermal growth factor, VEGF: Vascular Endothelial Growth Factor, RTKs: Tyrosine kninase receptors, NPR: Natriuretic peptide receptor, PI3K: Phosphoinositide 3-kinase, NO: Nitric Oxide, eNOS: edothelial Nitric Oxide, sGC: soluble Guanylyl cyclase, cGMP: cyclic guanylyl mono phosphate, SIRT1: Sirtuin 1, LKB1: Liver Kinase B1, PGC-1α: Peroxisome proliferator-activated receptor gamma coactivator 1-alpha, NAD^+^/NADH: Nicotinamide Adenine Dinucleotide/Nicotinamide Adenine Dinucleotide Phosphate, AMPK: Adenosine Monophosphate-activated Protein Kinase, ATP/ADP: Adenosine Triphosphate/Adenosine Diphosphate, CaMK: Calcium/calmodulin-dependent protein kinase, p38MAPK: p38 mitogen-activated protein kinases, CREB: cAMP-responsive element binding protein, ATF2: Activating transcription factor 2, CRE: cyclic AMP Response Element, MFF2: Myocyte Enhancer Factor-2, NRF1: Nuclear Respiratory Factor 1, Sp1: specificity protein 1, PPAR-γ/EERs: Peroxisome Proliferator Activated Receptor Gamma/Estrogen-Related Receptors, TFAM: Mitochondrial Transcription Factor A, TAZ: transcriptional coactivator with PDZ-binding motif, Rheb1/Rhebl1: Ras Homolog Enriched in Brain 1/Ras Homolog Enriched in Brain-like 1, mTOR: mammalian target of rapamycin, TOM/TIM: Translocons of the Outer/Inner membrane, mt: mitochondrial.

Mitochondrial biogenesis (MB) occurs in healthy eukaryotic cells to generate new mitochondria and increase the number of mitochondria via dividing the pre-existing ones, which already fully grow and their DNA replication is fully completed [22]. This process involves the expression of more than one thousand genes from both mitochondrial and nuclear genomes [23] and coordinates with cell cycle events but is not limited to cell division only [24]. Peroxisome proliferator-activated receptor γ (PPARγ) coactivator 1-α (PGC-1α) plays as the master regulator of MB. As shown in Figure 1, after activated via either phosphorylation or deacetylation by various signaling pathways such as receptor tyrosine kinases, natriuretic peptide receptors, and nitric oxide through cyclic guanosine monophosphate (cGMP), and cyclic adenosine monophosphate (cAMP), PPAR, Akt, sirtuin 1 (SIRT1)-mediated deacetylation, AMP/ATP via cyclic adenosine monophosphate (cAMP), AMP-activated protein kinase (AMPK), calcineurin/calmodulin, and specificity protein 1 (Sp1) [25,26], PGC-1α initiates MB pathway by sequential activations of nuclear transcription factors including nuclear respiratory factors 1, 2 (NRF1, NRF2), and estrogen-related receptor-α (ERR-α), followed by the increase in the expression mitochondrial transcription factor A (TFAM), then mitochondrial DNA (mtDNA) replication and transcription [27,28]. Translating mtDNA-encoded genes requires specific translation factors encoded by nuclear DNA, such as the initiation factors 2 and 3 (mtIF2 and mtIF3), elongation factors Tu, Ts, G1 (mtEFTu, mtEFTs, mtEFG1). NRF1 and NRF2, together with Sp1, bind to the gene of mitochondrial transcription factor A (TFAM) and promote the expression of TFAM, which is then imported via the translocase of the outer and inner mitochondrial membrane into mitochondria, where it regulates 13 mitochondrial genes encoding for essential components of ETC [26,29,30]. The mitochondrial proteins encoded by nuclear DNA are transported into the mitochondria via the translocase of the outer mitochondrial membrane/the translocase of the inner complex (TOM/TIM) (Figure 1). The regulation of MB is not limited to controlling PGC-1α activation through post-translational modifications but also PGC-1α transcription. Various signalings, including Akt, p38MAPK, Calcineurin A (CnA), calmodulin-dependent protein kinase IV (CamKIV), and protein kinase A (PKA) [31], as illustrated in Figure 1.

Among the PGC-1α activators, AMPK and SIRT1, the two metabolic sensors of the cells, are the two major pathways regulating mitochondrial biogenesis [32]. AMPK is activated as a response to different conditions that deplete cellular energy, including endurance exercise, hypoxia, starvation (primarily glucose), and mitochondrial electron transport chain (ETC) inhibitors [33,34,35,36,37,38]. AMPK either directly phosphorylates PGC-1α [39,40] or activates SIRT1 (an NAD^+^-dependent histone/protein deacetylase) through nicotinamide phosphoribosyl transferase which in turn increases NAD^+^ levels [41,42]. In contrast, SIRT1 also plays a role in AMPK activation [43]. SIRT1 activates AMPK through the deacetylation of Liver Kinase B1 (LKB1), the master upstream kinase, directly phosphorylating and activating AMPK and various related kinases [44,45]. A high NAD^+^/NADH ratio leads to the phosphorylation of SIRT1 at thr-522 residue. Phosphorylated SIRT1 activates PGC-1α by removing the acetyl group on it. Furthermore, the MB is initiated in response to cellular stress, such as oxidative stimulus, to an increase in the energy requirements of the cells [46] and external stimuli, including nutrients, hormones, and exercise [47,48,49]. As a result, MB increases the number of healthy mitochondria, resulting in a greater metabolic capacity to match the cell’s energy needs.

A recent study discovered a novel stimulator of MB called a transcriptional coactivator with PDZ-binding motif (TAZ) [50]. PGC-1α and TAZ act at different levels in TFAM production. PGC-1α induces *Tfam* gene transcription. Meanwhile, TAZ promotes the translation of *Tfam* mRNA via Ras homolog enriched in brain (Rheb)/Rheb-like-1 (Rhebl1)- Mammalian target of rapamycin complex 1 (mTORC1) axis. TAZ also plays a vital role in the translational regulation of other mitochondrial genes [27,50,51]. Moreover, in skeletal myocytes derived from muscle-specific TAZ-knockout (mKO) mice, the Tfam protein level is decreased and not induced after exercise. However, there is no change in PGC1α, NRF1, or NRF2 levels [50].

### 2.2. Mitochondrial Transport

**Figure 2:**
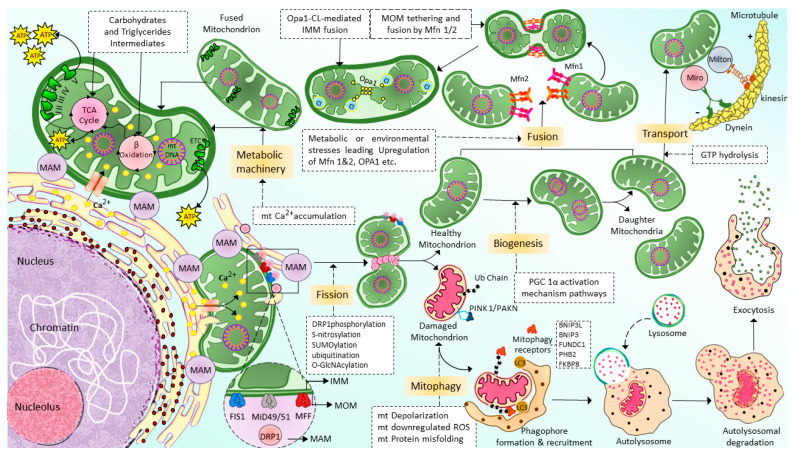
Schematic of the machinery and context of mitochondrial dynamics: Fission, fusion, transport, and mitophagy. Mitochondria generated from mitochondrial biogenesis are transported and distributed through the cells via cytoskeletal elements, including actin microfilaments and microtubules by kinesin and dynein motors. Mitochondrial fusion is a highly regulated and complex process in which two mitochondria are fused into one mitochondrion to maximize the OXPHOS capacity of the cells under conditions of high energy demand. Mfn1/2 is responsible for the fusion of MOMs; meanwhile, Opa1 is responsible for the fusion of IMMs. In contrast to fusion, mitochondrial fission allows a mitochondrion to segregate into two heterogenous mitochondria and enable autophagy removal of the damaged mitochondrial. Drp-1 is the primary protein that is responsible for mitochondrial fission. However, additional proteins might also be necessary for this process, such as FIS1, MFF, and MiD49/51. Finally, the damaged, defective mitochondria generated by mitochondria fission are eliminated via a selective autophagy process known as mitophagy. (For more details, please see the main text). TCA: tricarboxylic acid, MAM: mitochondria-associated ER membrane, Ca^2+^: calcium, mt: mitochondrial, ETC: electron transport chain, ATP: adenosine triphosphate, GTP: guanylyl triphosphate, Opa1: optic atrophy 1, Mfn1/2: mitofusin 1/2, Drp1: dynamin-related protein 1, FIS1: mitochondrial fission 1 protein, MFF: mitochondrial fission factor, and MiD49/51: mitochondrial dynamin protein 49/51, MOM: mitochondrial outer membrane, IMM: inner mitochondrial membrane, ROS: reactive oxygen species, ub: ubiquitination, PINK1/PAKN: PTEN-induced putative kinase protein 1/Parkin-mediated ubiquitination, BNIP3L: BCL2/adenovirus E1B 19 kDa protein-interacting protein 3-like, BCL2/adenovirus E1B 19 kDa protein-interacting protein 3, FUNDC1: FUN14 domain containing 1, PHB2: Prohibitin-2, FKBP8: FK506-binding protein 8.

Mitochondrial transport through the cytoskeleton is crucial for normal mitochondrial morphology, network, motility, and distribution [52]. The cytoskeletal elements, actin microfilaments, and microtubules are necessary to transport and distribute mitochondria. The motor-based mitochondrial movement allows the mitochondria to navigate along microfilaments and tubules. Mitochondria bind to specific motor isoforms through adaptors explicitly designed for each organelle. In order to act as receptors and localize the motor adaptor complex to the organelle, adaptors probably need integrated outer mitochondrial membrane proteins. Thus, adaptor proteins that combine with the motors set up organelle-specific connections. Alteration of these motor and adapter proteins can impair the mitochondrial transport process. In multicellular eukaryotes, mitochondria use microtubule motors, such as the kinesin/dynein motor (Figure 2), which consists of plus-end-directed kinesins and minus-end-directed dyneins [53,54].

### 2.3. Mitochondrial Fusion 

Mitochondrial fusion is a complex regulatory mechanism involving several proteins’ binding from multiple mitochondrion membranes (Figure 2). When cells experience metabolic or environmental stress, mitochondria undergo fusion by cointegrating partially damaged mitochondria, lowering the stress by optimizing ATP production [55]. The outer membrane (MOM) of the mitochondria contains mitochondrial fusion factor-mitofusins, a member of the hydrolase enzyme family. Mitofusins are essential for the fusion of the MOM. The ocular atrophy protein 1 (OPA1), anchored to the inner mitochondrial membrane (IMM), allows the IMM to undergo fusion. The cristae organization is necessary to produce cellular energy, the mitochondrial ETC, mitochondrial membrane potential (ΔΨm) generated by proton pumps, regulation of cell apoptosis, and maintenance of mtDNA are modulated by OPA1 [56]. MFN1, MFN2, and OPA1 join the inner and outer membranes of mitochondria during the fusion mechanism. In certain cellular scenarios, a functional substitution between MFN1 and MFN2 occurs. This is brought on by cells lacking MFN1 inducing MFN2 to overexpress and vice versa [57]. There is a significant relationship between cellular physiological activity and mitochondrial fusion. During the fusion mechanism, the old mitochondrial transfers proteins and mtDNA to newly formed mitochondria, which in turn assist in limiting the accumulation of damaged mtDNA [58]. Due to ROS-induced mutations in mtDNA [59], some mitochondria are unable to perform their respiratory activity optimally in order to improve the fusing mitochondria’s overall respiratory function. Therefore, mitochondrial fusion is essential because it enables the interchange of gene products and metabolites. Similarly, diminished mitochondrial function is linked to a restriction of mitochondrial fusion [60]. Inhibition of fusion impairs OXPHOS, induces mtDNA depletion, and elevates ROS production [61]. The disruption of the fission mechanism causes imbalanced fusion, leading to an increase in the number of elongated mitochondria. However, interference with the fusion process leads to more fragmented mitochondria [62].

### 2.4. Mitochondrial Fission

Mitochondrial fission segregates a mitochondrion into two heterogenous mitochondria without mDNA (Mitochondrial DNA) replication (Figure 2). Fission is crucial for removing damaged organelles by mitophagy. Mitochondrial division, size, and form, as well as the distribution of mitochondria throughout the cell, depending on mitochondrial fission. There are two major ways that mitochondria undergo fission: Mitochondrial Fission Process 1 (MTFP1), in which the IMM splits first and eventually causes the separation of OMM, and Mitochondrial Fission Process 2 (MTFP2), in which the mitochondria accumulate from the fission point inward and separate. Dynamin-related protein 1 (DRP1), a GTPase fission mediator, is recruited from the cytosol and proceeds to the MOM, forming foci and rings surrounding mitochondria to mediate the fission process. Currently, most of the proteins which are known to be associated with mitochondrial fission are GTPase family proteins, including DRP1 itself and others such as mitochondrial fission 1 protein (FIS1), mitochondrial fission factor (MFF), and mitochondrial dynamics proteins of 49 and 51 kDa (MiD49 and MiD51). MiD49 and MiD51 are anchored in the MOM surface [63,64,65,66]. The structure of the DRP1 includes an N-terminal GTPase domain followed by the middle domain, variable domain (or Binsert), and the GED in the C-terminal. A critical mechanistic process of systolic Drp1 recruitment into the MOM surface is due to the proposed act as receptors in the MOM. The MOM receptors in this process are FIS1, MFF, MiD49, and MiD51 [67,68].

### 2.5. Mitochondrial Selective Autophagy (Mitophagy)

Mitophagy [69], also known as mitochondrial selective autophagy [70], is crucial for reestablishing cellular balance in both normal physiology and during stressful circumstances [71]. Mitophagy is a process that eliminates the damaged or extra mitochondria as double-membraned autophagosomes for subsequent lysosomal degradation. Mitochondrial fragmentation by fission is one of the crucial processes associated with mitophagy [72,73]. Hence, mitophagy and mitochondrial fission regulate the abundance of mitochondria and their functionality in the host cells [74]. Mitophagy is a multi-stage process that involves numerous phases (Figure 2). They can be categorized as follows: mitophagy initiation phase, mitochondrial labeling for autophagy machinery detection, production of autophagosomes for labeled mitochondria engulfment, lysosomal trapping, and enzyme hydrolysis [75]. Multiple regulatory mitophagy mechanisms are currently induced in response to various stimuli. They are typically divided into two categories: receptor-mediated mitophagy and PTEN-induced putative kinase protein 1 (PINK1)/Parkin-mediated ubiquitination (PAKN) mitophagy pathway [76]. In response to various mitochondrial stressors, receptor-mediated mitophagy is controlled at the transcriptional or post-transcriptional level [70]. The mitophagy receptors residing in the MOM can attract autophagosomes to mitochondria. This is the main characteristic feature of mitophagy receptors [77,78]. BCL2 Interacting Protein 3-like (BNIP3L or NIX), BCL2 interacting protein 3 (BNIP3), FUN14 domain containing 1 (FUNDC1), Prohibitin2 (PHB2), and FKBP Prolyl Isomerase 8 (FKBP8) are the few examples of mitophagy. At least one LC3 interacting region (LIR), which can directly bind to the autophagy mediator LC3 and attract autophagosomes to mitochondria, is a characteristic of mitophagy receptors [79].

The most well-known mitophagy pathway that controls mitochondrial maintenance and quality control is the PINK1/PAKN mitophagy pathway [71]. PINK1 is a serine/threonine kinase with a mitochondrial target sequence (MTS) and transmembrane domain (TMD), and PAKN is a cytosol ubiquitin E3 ligase [74]. Together, PINK1/PAKN detects cellular stress and coordinates the elimination of damaged mitochondria. PINK1 is continuously imported into mitochondria under physiological circumstances with normal mitochondrial membrane potential, where it is cleaved by the intramembrane protease presenilin-associated rhomboid-like (PARL) protein, causing its retro-translocation into the cytosol and quick proteasomal degradation [80,81].

## 3. Mitochondrial Dynamics and Metabolism in the Pathophysiology of T2DM

### 3.1. Mitochondrial Dynamics in T2DM

T2DM and its vascular complications are associated with mitochondrial dynamics. Mitochondria acts as an important regulator of insulin secretion. In addition, Mitochondria are the primary generators of ROS and play a central role in cellular apoptosis and cell death. High glucose levels in T2DM increase ROS production [82,83,84], promoting oxidative stress and activating various signalings mediating the micro and macrovascular complications in diabetes [83,85,86]. High-calorie intake causes intramyocellular lipid accumulation [87,88,89], enhancing ROS production, oxidative stress, and mitochondrial dysfunction in the skeletal muscle [90,91,92]. Obesity disrupts the β-oxidation of FA into acetyl-CoA to supply the substrates for utilization in the TCA [93]. Decreased β-oxidation causes cellular dysfunction due to the synthesis of triglycerol and ectopic deposition of lipids. Increased free FA also promotes ROS production resulting from the increase in lipid peroxidation byproducts [91,94], the effect known as lipotoxicity. The activities of antioxidant enzymes such as glutathione S-transferase, glutathione peroxidase, paraoxonase-1, and catalase decrease, which, therefore, superimpose the oxidative stress condition in different tissues of obese rats [95]. Therefore, ROS is an important mediator in dysregulated mitochondrial function and T2DM. The changes in mitochondrial morphology and dynamics are required to increase ROS under hyperglycemia [96]. Therefore, mitochondrial dynamics are a major regulator of mitochondrial function. Skeletal muscle derived from obesity and T2D individuals have an impaired functional capacity of mitochondria correlated with decreased mitochondrial size, number, and damaged cristae [96,97]. Hyperglycemia also induces the disruption of mitochondria in other cell types, including the heart, vascular, and liver [96,98,99]. However, glucose metabolic disorders do not always play the causal factor; it might also be the dysregulated mitochondrial dynamics [100,101,102], as discussed later.

**Figure 3:**
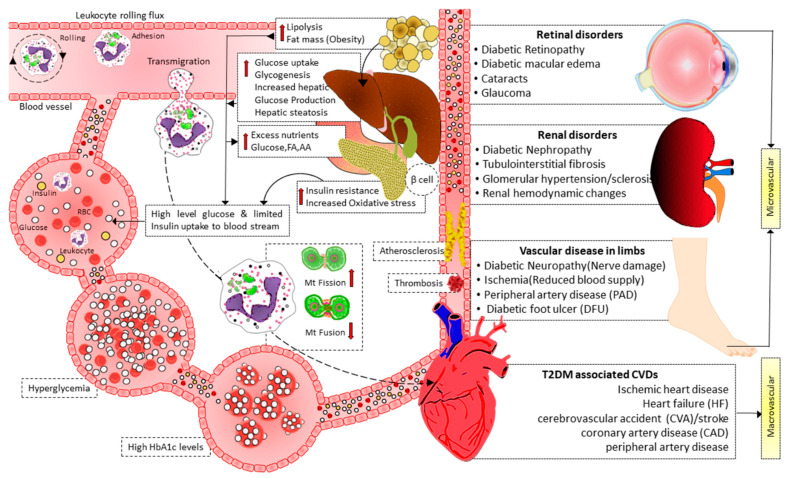
Illustration showing the interplay of mitochondrial dynamics in activating leukocyte–endothelial contacts and micro and macrovascular complications of diabetes. Hyperglycemia in T2D induces mitochondrial fission and decreases mitochondrial fusion, facilitating leukocyte activation and endothelial interactions. Furthermore, high blood glucose levels also cause mitochondrial fission, impairing endothelial function via activating iNOS and increasing ROS production. As a result of leukocyte activation, leukocyte–endothelial interactions, endothelial dysfunction, atherosclerosis, and thrombosis mediate micro- and macro-vascular complications in T2D. FA: fatty acid, AA: amino acid, Mt: mitochondrial, HbA1c: hemoglobin A1c, T2D: type 2 diabetes, CVDs: cardiovascular diseases.

Insulin signaling promotes the expression of Opa-1 protein via the Akt-mTOR-NFkB-Opa-1 pathway, leading to mitochondrial fusion [103]. In T2DM, this signaling is disrupted. Therefore Opa-1 expression level decreases, leading to imbalanced fission, reducing ATP production efficiency, and causing cellular dysfunction. Reduced MFN2 expression is associated with T2DM and may be connected to skeletal muscle mitochondrial dysfunction. Furthermore, it has recently been shown that in leukocytes from T2D patients, mitochondrial fusion was reduced, and fission was enhanced, especially in patients with poor glycemic control [104]. In addition, the activation of leukocyte–endothelial contacts in diabetes patients was associated with lower mitochondrial fusion and higher mitochondrial fission. Leukocyte rolling flux also increased concurrently with HbA1c levels [104]. These findings imply that glycemic management affects mitochondrial dynamics in the leukocytes of diabetic patients, where there is an increase in leukocyte–endothelial contacts and a decrease in mitochondrial fusion (Figure 3). Therefore, it is theorized that having T2D with poor glycemic management changes mitochondrial dynamics, encouraging leukocyte–endothelial interactions and the development of cardiovascular diseases and other vascular complications [103,104]. Moreover, hyperglycemia impaired endothelial function directly by promoting mitochondrial fission, iNOS activation, and ROS production [105].

White adipose tissue derived from *ob/ob* mice (obese mice model) exhibit a fusion-to-fission balance reflected by the increase in Drp-1 and the decrease in MFN2 and OPA1 protein expression, decreased mitochondrial biogenesis evidenced by a reduced content of mitochondrial DNA and PGC-1α mRNA expression. Treatment with either leptin or mitochondrial division inhibitor (mdivi-1) improved blood glucose levels, lipid oxidation, and mitochondrial function by storing mitochondrial dynamics balance [106].

Moreover, T2D dyslipidemia models exhibit elevated mitochondrial fission due to mitochondrial depolarization, decreased ATP synthesis, elevated oxidative stress, and decreased insulin-stimulated glucose uptake. Insulin resistance and mitochondrial fragmentation brought on by excessive palmitate were reduced by both genetic and pharmaceutical suppression of DRP1 [107]. In another study, DRP1 was elevated in rat pancreatic islets after stimulation by FFAs, and this DRP-1 overexpression was accompanied by increased β-cell death [108]. Many processes involved in atherosclerosis (a common complication of T2D) are linked to mitochondrial fission, including endothelial dysfunction [105,109,110,111,112,113], collagen matrix [114,115], and the motility and proliferation of vascular smooth muscle cells [116,117,118,119]. In endothelial cells derived from T2D patients, silencing FIS1 or DRP1 inhibited high glucose-induced mitochondrial fission and ROS generation [105]. Metformin prevents the DRP1-mediated mitochondrial fission that leads to atherosclerosis in diabetic mice via an AMP-activated protein kinase (AMPK)-dependent pathway [120]. DRP1 is essential in the pathogenesis of diabetic micro and macrovascular complications [121,122,123,124,125,126,127,128,129,130,131,132,133,134,135]. 

Metabolic disorders affect mitochondrial fusion/fission balance and impair biogenesis [136,137,138,139]. Downregulation of PGC-1α (the master regulator of metabolism and mitochondrial biogenesis) leads to mitochondrial damage and decreased mitochondrial density in obesity. The serum concentrations of adiponectin were reduced in obese subjects. The adiponectin can stimulate deacetylation and transcriptional activity of PGC-1α and MB via engaging to adiponectin receptor to activate Ca^2+^ and AMPK/SIRT1 signaling [140]. Therefore, the downregulation of PGC-1α gene expression or activity is implicated in obesity and diabetes [141,142,143]. Physical exercise can improve insulin resistance by restoring PGC-1α activation and expression; hence, MB is mediated by increased intracellular Ca^2+^ and AMPK/SIRT1 signaling (Figure 1). 

### 3.2. Dysregulated Mitochondrial Dynamics and Metabolism Play a Causal Role in Diabetes

**Figure 4:**
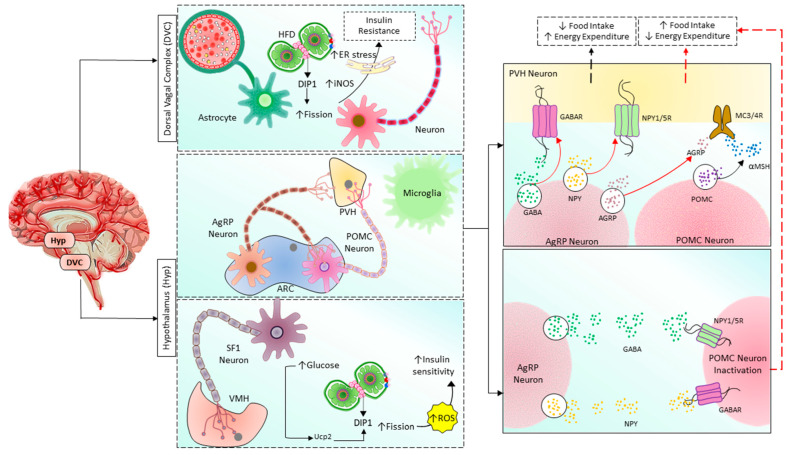
Mitochondrial fusion/fission balance plays a vital role in regulating the body’s metabolism by the brain in the hypothalamus and DVC. Hypothalamus has two nuclei involving energy homeostasis: ARC and VMH. By controlling PVH neurons, POMC and AgRP neurons regulate food intake and energy expenditure. POMC neurons release POMC, which is then cleaved into α-MSH and stimulates VMH neurons via MC3/4R, finally promoting negative energy balance by inhibiting food intake and increasing energy expenditure. In contrast, AgRP neurons directly inhibit VMH neurons via releasing AgRP, GABA, and NPY, acting on MC3/4R, GABAR, and NPY1/5R, respectively, finally promoting positive energy balance by increasing food intake and reducing energy expenditure. AgRP neurons also indirectly promote positive energy balance by inhibiting POMC neurons through GABAR and NPY1/5R. In addition, in SF1 neurons of VMH, increased glucose level induces mitochondrial fission followed by ROS production and insulin resistance in a upc2-dependent manner. On the other hand, excessive nutrients in the astrocytes and neurons located in DVC cause insulin resistance by increasing mitochondrial fission, iNOS activity, and ER stress. HFD: high-fat diet, ER: endoplasmic reticulum, Drp1: dynamin-related protein 1, iNOS: inducible nitric oxide synthase, AgRP: Agouti-related peptide, POMC: Pro-opiomelanocortin, PVH: paraventricular nucleus of the hypothalamus, ARC: arcuate nucleus, SF1: steroidogenic factor-1, ROS: reactive oxygen species, GABA: Gamma-aminobutyric acid, GABAR: GABA receptor, NPY: neuropeptide Y, NPY1/5R: NPY receptor 1/5, α-MSH: α-melanocyte-stimulating hormone, MC3/4R: melanocortin-3/4 receptor.

**Figure 5:**
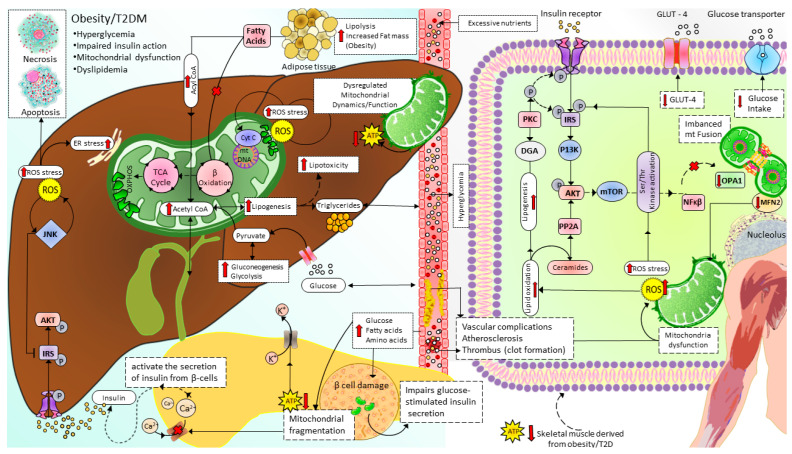
Mitochondrial dynamics and metabolism in the pathophysiology of T2D. Excessive nutrients (for example, HDF) induce imbalanced mitochondrial dynamics leading to decreased OXPHOS capacity and accumulation of FA and lipotoxicity in which ceramides and DAG impair insulin signaling. In addition, reduced ATP/ADP ratio in pancreatic β-cells disrupts glucose-stimulated insulin secretion, further increasing blood glucose levels. T2D: type 2 diabetes, ROS: Reactive oxygen species, IRS: Insulin receptor substrate signaling, AKT: serine/threonine kinase pathway, JNK: c-junN-terminal kinase pathway, ER: endoplasmic reticulum, TCA cycle: tricarboxylic acid cycle, ATP: adenosine triphosphate, Ca^2+^:calcium ion, β oxidation: beta-oxidation of fatty acid (Fatty acid cycle), GLUT4: glucose transporter type 4, P13K: phosphatidylinositol-3 kinase, PP2A: protein phosphatase 2A, PKC: protein kinase C, DGA: diacylglycerol, mTOR: mammalian target of rapamycin, NFKβ: nuclear factor kappa light chain enhancer of activated beta cells, OPA1: optic atrophy-1, MFN2: mitofusin2.

Mitochondrial dysfunction due to imbalanced mitochondrial dynamics leads to decreased OXPHOS, ATP production, β-oxidation of FFA [97,144], and enhanced ROS production [145]. Mitochondrial dysfunction plays a causal role by governing insulin secretion failure in β-cells and peripheral insulin resistance through the gluco-lipotoxicity [5,6] (Figure 4) or by impairing the regulation of glucose homeostasis in neurons of different cerebral regions [92,146,147,148,149,150] and glial cells in the brain (Figure 5). Excessive energy supply and low ATP demand increase mitochondrial fission, proton leak, and redox imbalance. On the other hand, in the depletion of nutrient supply and high ATP demand, the mitochondria are hyperfused, and respiration is optimized [151]. Accumulation of lipids/FFA increases diacylglycerol (DAG) and ceramides which activate protein kinase C (PKC) and protein phosphatase 2 (PP2A), respectively. PKC phosphorylates and inhibits insulin receptors. Meanwhile, PP2A dephosphorylates Akt and impairs insulin’s downstream signaling in which glucose transporter translocates to the cell membrane [152,153,154]. Furthermore, increased oxidative stress induces mitophagy and apoptosis, exacerbating the decrease in β-oxidation of FFA further, and ROS promotes Serine/threonine-specific protein kinases (Ser/Thr kinases) which phosphorylate and inhibit insulin receptor substrate (IRS) [155,156]. All these effects finally cause insulin resistance [157].

Chronic exposure to high levels of glucose, FA, and amino acids damages β-cells. It impairs glucose-stimulated insulin secretion due to mitochondrial fragmentation, which decreases the production of ATP (increased ATP/ADP ratio close ATP-sensitive K^+^ channels and depolarization of membrane potential initiates, followed by increased calcium levels which activate the secretion of insulin from β-cells) [158]. 

The previous study in cybrid b4 discovered that overexpression of MFN1/2 or DRP1/FIS1 knockdown increased mitochondrial fusion, insulin signaling, and Glu4 translocation to membranes [159]. In contrast, MFN1/2 knockdown or DRP1/FIS1 overexpression reduced the mitochondrial network (promoted fission) and decreased IRS-AKT signaling and Glut4 translocation [159]. Furthermore, a later study reported that excessive nutrients also increased chronic inflammatory markers, inflammasome, and these effects were abolished by overexpression of fusion proteins (Mfn1 or Mfn2) [160]. In another study on L6 rat-derived skeletal muscle cells, silencing MFN2 and OPA1 induced mitochondrial fragmentation, attenuation of cellular respiration, and insulin-dependent Akt phosphorylation [161]. The results from this study suggest that the imbalance of mitochondrial fusion and fission plays a causal role in the pathogenesis of insulin resistance and T2DM.

In mammals, the hypothalamus and dorsal vagal complex (DVC) have been implicated as crucial regulators of whole-body metabolism [149,150,162]. In the hypothalamic arcuate nucleus, there are populations of neurons known as arcuate melanocortin neurons that regulate the body’s metabolism. The first group secretes agouti-related peptide (Agrp) and neuropeptide-Y (NPY), which promote food intake and decrease energy expenditure [163,164,165,166,167]. These neurons’ activity increases when the body’s energy is depleted [168,169,170,171,172]. The second group is the pro-opiomelanocortin (POMC)-expressing neurons that function oppositely in regulating metabolism. The difference in functions of these two neuronal populations is due to the opposite effects on their target neurons, the melanocortin 3/4 receptor (MC3/4R)-expressing neurons in the paraventricular nucleus of the hypothalamus (PVH) (Figure 4). POMC neurons release α-melanocyte stimulating hormone (α-SMH, a cleaved product of POMC), which binds to and activates MC4R in the paraventricular nucleus of the hypothalamus to inhibit food intake and promote energy expenditure [173]. AgRP neurons release AgRP, which inhibits MC3/4R signaling, stimulating food intake and reducing energy expenditure [164,166,167,174]. Interestingly, these neurons also release NPY and neurotransmitter gamma-aminobutyric acid (GABA), which inhibit POMC neurons via neuropeptide Y receptor 1 and 5 (NPY1R and NPY5R) and GABA_A_ receptors, respectively [175,176,177,178,179]. Therefore, once activated, AgRP neurons can amplify their effects by inhibiting their antagonizing neurons. The energy state reciprocally regulates POMC and AgRP neurons, the adiposity hormones leptin, and insulin. AgRP neurons are inhibited by leptin and insulin [180,181,182,183] and activated by the gastric hormone ghrelin [184,185,186]. In contrast, leptin and insulin activate POMC neuron signals under high energy demand [175,187,188,189,190,191]. Neurons require high energy relative to other cells, they utilize 20% of whole-body oxygen consumption [192], and mitochondria are the primary factory that generates and supplies ATP to them. Therefore, the mitochondria have a critical role in maintaining neuronal activities. Mitochondrial functions are reflected by their morphology and dynamics, which can be changed to support cellular energy homeostasis in response to different conditions. As mentioned above, the balance of mitochondrial dynamics in neurons supports cellular function and neuronal activities to control energy homeostasis [193]. Schneeberger et al. found that POMC-specific Mfn knockout mice fed the HF diet had fewer mitochondrial-ER contacts, decreased mitochondrial length, and branching in POMC neurons [150]. Specific MFN2 knockout in these neurons decreased α-MSH release, altered mitochondrial morphology, decreased mitochondrial-ER contacts and ER stress, reduced the conversion of POMC into α-MSH, developed leptin resistance due to ER stress, increased ROS production due to impaired complex I activity, increased energy intake, decreased energy consumption, and finally lead to obesity [150]. These effects can be reversed by chemical chaperones such as 4-phenyl butyric acid (4-PBA) or tauroursodeoxycholic acid (TUDCA), which relieves ER stress [150]. These results suggested that MFN2 in POMC neurons is the key regulator of body energy homeostasis via mitochondrial-ER axis homeostasis and function. Surprisingly, POMC-specific OPA1-knockout mice develop obesity at 7 weeks of age and exhibited mitochondrial cristae loss fragmentation, Ca^2+^ overload, and decreased α-MSH release, but glucose sensing, neuronal activation, and hypothalamic ROS production are not changed [148,194]. DRP1-mediated mitochondrial fission negatively regulates POMC neuronal responses to glucose and leptin sensitivity contributing to obesity and diabetes development. Selective Drp1 knockout in POMC neurons inhibits mitochondrial fission and improves glucose metabolism and leptin sensitivity [195]. More detailed insights into the role of mitochondrial dynamics in the hypothalamic regulation of glucose and energy homeostasis can be found in Sungho Jin and Sabrina Diano’s review [196].

Recent studies also found that under excessive nutrients, Drp1 is activated, mediates mitochondrial fission in DVC neurons, and induces insulin resistance [92]. Inhibition of Drp1 by infusion of mitochondrial division inhibitor 1 (MDIVI-1) reverses HFD-induced mitochondrial fragmentation, endoplasmic reticulum (ER) stress, and insulin resistance in DVC neurons. Furthermore, activation of Drp1 alone is sufficient to cause mitochondrial fission, ER stress in DVC neurons, and insulin resistance. ER stress is the critical mediator in mitochondrial fission-induced insulin resistance in DVC, and relief of ER stress using 4-PBA restores insulin sensitivity in DVC of 3-day HFD rats [92]. Moreover, in a recent study, Patel et al. found that mitochondrial dynamics in DVC astrocytes also play an important role in whole-body metabolism. Increased Drp1 activity disrupts insulin signaling, increasing food intake, weight gain, and adipose tissue. Drp1 activation also increases DVC’s inducible nitric oxide synthase (iNOS) levels (Figure 4). Either inhibition of Drp1 or iNOS can prevent HFD-induced insulin resistance and obesity [197]. Astrocyte-specific conditional deletion of MFN2 suppressed perivascular mitochondrial clustering and disrupted mitochondria-endoplasmic reticulum (ER) contact sites. These results suggest that mitochondrial fission mediated by Drp1 and ER stress are essential in regulating whole-body energy homeostasis of DVC.

The activation of Drp1 and mitochondrial fission requires the functional integration between phosphorylations at S600 and at S579 sites of Drp1 [19]. S600 phosphorylation occurs first, then initiates the phosphorylation of the other one. The mice expressed S600 phosphor null form (S600A, which interferes with the phosphorylation of S600) have decreased mitochondrial fission, increased lipid oxidation and OXPHOS capacity, insulin sensitivity, and thermogenic response under HFD feeding [19]. In addition, phosphorylation at S616 promotes mitochondrial fission [198], but phosphorylation of Drp1 at S637 causes the opposite effect on mitochondrial dynamics [199,200]. Therefore, post-translational modifications of Drp1 are important in regulating mitochondrial fission. Moreover, Syntaxin 4, an exocytosis protein, enhances glucose uptake and improves insulin sensitivity in HFD diabetic mice by suppressing mitochondrial fission through phosphorylation of Drp1 at S637 in a pathway involved in AMPK [201].

In response to energy depletion due to decreased food intake or increased demand during exercise, mitochondrial biogenesis is activated to maintain and improve the number of healthy mitochondria for ATP production [50,202,203,204,205,206,207]. The disruption of mitochondrial biogenesis decreases OXPHOS and ATP production capacity and is associated with metabolic disorders, including obesity and T2DM [197,208]. PGC-1α is the central inducer of mitochondrial biogenesis [209,210] and is a potential target for modulating mitochondrial mass. The conditions of energy depletion, such as endurance exercise, activate PGC-1α activity through Ca^2+^/CaMK/calcineurin, AMPK/SIRT1, NO, and Akt signalings and enhance PGC-1α transcription through CaMK, calcineurin, and Akt pathways (Figure 1). In skeletal muscle during the aging of mice, Tina Wenz et al. found that PGC-1α overexpression preserved mitochondrial function, neuromuscular junction, and muscle integrity, reduced ROS production, apoptosis, autophagy, proteasome degradation, and improved insulin sensitivity in skeletal muscle of mice during aging [211]. Recent compelling evidence has shown that exercise promotes MB by activating PGC-1α [212,213,214,215] and increases mitochondrial function in human skeletal muscle [47,216,217,218,219,220]. Jun-Ha Hwang et al. recently discovered the underlying mechanism that exercises grows MB via transcriptional coactivator with PDZ-binding motif (TAZ), which induces TFAM transcription through Ras homolog enriched in brain (Rheb)/Rheb-like-1 (Rhebl1)-mTOR axis. Therefore, TAZ is responsible for MB and exercise-induced muscle adaption [50]. In alloxan, monohydrate-induced diabetic rabbits, pioglitazone (an anti-diabetic drug used to treat T2DM) improves mitochondrial biogenesis and function and reduces NF-κB and TGF-β1 expression levels via the PPAR-γ/PGC-1α pathway. These effects of pioglitazone improve diabetic atrial structural and electrophysiological remodeling. When PGC-1α is silenced using siRNA transfection, the impacts of pioglitazone are blunted [137]. These results again suggest that PGC-1α is the efficient target in regulating mitochondrial dynamics, hence mitochondrial function and metabolic homeostasis in T2DM.

ROS is involved in various pathological processes, especially in mitochondrial dysfunction and apoptosis. In the mice model, ROS activates nuclear factor-κB (NF-κB), stimulating cytokine production (tumor necrosis factor-α, interleukin 6), leading to increased lipolysis, nonesterified fatty acid, glycerol levels, gluconeogenesis, and de novo lipogenesis, finally develop insulin resistance and diabetes. In addition, NF-κB also causes p62 protein/sequestosome 1 (p62/SQSTM1) accumulation, recruiting damaged mitochondria with polyubiquitin chains and thereby inducing excessive mitophagy. These effects of ROS are ameliorated by the administration of ROS scavenger or NF-κB inhibitor [221].

## 4. Therapeutic Opportunity of HDAC Inhibition for Mitochondrial Dysfunction in DM

Histone deacetylase (HDACs), one of the critical epigenetic regulators, has been shown to improve multiple metabolic disorders and regulate mitochondrial biogenesis and function. There are 18 mammalian HDAC isoforms that were identified and classified into four classes according to their sequence homology with yeast. The catalytic activity of classes I, II, and IV HDACs requires cofactor zinc ions, whereas class III HDACs are NAD^+^-dependent. Class I HDACs (HDAC1, HDAC2, HDAC3, and HDAC8) are ubiquitously distributed in the nucleus. Class II is further subdivided into class IIa (HDAC4, HDAC5, HDAC7, and HDAC9) and class IIb (HDAC6 and HDAC10). Class IIa HDACs shuttle between the cytoplasm and the nucleus with minimal catalytic activity. HDACs were recently identified as a potential therapeutic target for diabetes [222,223,224]. HDACs play important roles in regulating insulin-mediated cell signaling. Transcriptional expression of Glut4 is mainly regulated by myocyte enhancer factor 2 (MEF2), which binds to the Glut4 promoter. HDAC5 can interact with MEF2 and act as a transcriptional repressor of Glut4 by deacetylating histones and compacting chromatin structure. This inhibitory complex is released once HDAC5 is phosphorylated by AMPK and CaMK, allowing HDAC5 shuttle to cytoplasm and release from the inhibitory complex, instead, recruiting coactivator PGC-1α to increase Glut4 transcription. AMPK activator and HDAC5 siRNA are reported to enhance the transcriptional expression of Glut4 in myotube cells and adipocytes [225,226]. Moreover, HDAC2 can bind to insulin receptor substrate 1 (IRS1) in the liver cells of obese animals. HDAC2 inhibition with TSA (a pan HDAC inhibitor) or siRNA-mediated knockdown attenuates IRS-1 deacetylation and partially restores insulin signaling [227].

HDACs also regulate mitochondrial dynamics via deacetylating fission and fusion machinery and biogenesis via decreased expression of the primary regulator of MB, PGC-1a. The pan HDAC inhibitor SAHA induces mitochondrial elongation via decreasing expression of mitochondrial fission protein Fis1 and reduces the translocation of Drp1 to the mitochondria in Hep3B cells [228]. TSA also prevents mitochondrial fragmentation and provides neuroprotection against triggers for mitochondrial fragmentation (MPP^+^) in dopaminergic neurons via inhibiting histone deacetylation of the Mfn2 promoter and avoiding transcriptional repression of Mfn2 [229]. Additionally, SAHA can upregulate PGC-1 expression in mice and cultured cardiomyocytes [230]. Therefore, HDACs play pathological regulatory roles in glucose uptake, insulin resistance, and mitochondrial dynamics. Accordingly, HDACs may thus be a target for the treatment of insulin resistance and dysregulated mitochondrial dynamics. HDACs also regulate inflammation, oxidative stress, fibrosis, cell cycle and death, cellular metabolism, and other pathological processes through catalyzing deacetylation of the histones and non-histone proteins [227]. Accordingly, HDAC inhibition is expected to be a novel therapeutic strategy for DM through its effects on mitochondrial dynamics and several biological activities.

## 5. Conclusions

Mitochondrial dynamics and metabolic disorders are commonly seen in overnutrition. Targeting mitochondrial dynamics (such as HDAC inhibition) via promoting mitochondrial biogenesis, restoration of mitochondrial fusion/fission balance, and mitophagy intervention are potential strategies for improving insulin resistance and T2DM. More clinical studies are needed to evaluate the efficacy of these interventions on human beings.

## Data Availability

Not applicable.
